# Adherence to Tuberculosis Therapy among Patients Receiving Home-Based Directly Observed Treatment: Evidence from the United Republic of Tanzania

**DOI:** 10.1371/journal.pone.0051828

**Published:** 2012-12-19

**Authors:** Abdallah Mkopi, Nyagosya Range, Fred Lwilla, Saidi Egwaga, Alexander Schulze, Eveline Geubbels, Frank van Leth

**Affiliations:** 1 Ifakara Health Institute, Dar es Salaam, Tanzania; 2 National Institute for Medical Research, Dar es Salaam, Tanzania; 3 National Tuberculosis and Leprosy Programme, Ministry of Health and Social Welfare, Dar es Salaam, Tanzania; 4 Novartis Foundation for Sustainable Development, Basel, Switzerland; 5 KNCV Tuberculosis Foundation, The Hague, The Netherlands; 6 Department of Global Health, Academic Medical Centre, University of Amsterdam, Amsterdam Institute for Global Health and Development, Amsterdam, The Netherlands; Uniformed Services University of Health Sciences, United States of America

## Abstract

**Background:**

Non-adherence to tuberculosis (TB) treatment is the leading contributor to the selection of drug-resistant strains of *Mycobacterium tuberculosis* and subsequent treatment failure. Tanzania introduced a TB Patient Centred Treatment (PCT) approach which gives new TB patients the choice between home-based treatment supervised by a treatment supporter of their own choice, and health facility–based treatment observed by a medical professional. The aim of this study was to assess the extent and determinants of adherence to anti-TB therapy in patients opting for home-based treatment under the novel PCT approach.

**Methods:**

In this cross-sectional study, the primary outcome was the percentage of patients adherent to TB therapy as detected by the presence of isoniazid in urine (IsoScreen assay). The primary analysis followed a non-inferiority approach in which adherence could not be lower than 75%. Logistic regression was used to examine the influence of potentially predictive factors.

**Results:**

A total of 651 new TB patients were included. Of these, 645 (99.1%) provided urine for testing and 617 patients (95.7%; 90%CI 94.3–96.9) showed a positive result. This result was statistically non-inferior to the postulated adherence level of 75% (p<0.001).

**Conclusions:**

Adherence to TB therapy under home-based Directly Observed Treatment can be ensured in programmatic settings. A reliable supply of medication and the careful selection of treatment supporters, who preferably live very close to the patient, are crucial success factors. Finally, we recommend a cohort study to assess the rate of adherence throughout the full course of TB treatment.

## Introduction

The Tanzanian National Tuberculosis and Leprosy Control Programme (NTLP) changed its first-line treatment regimen for new cases of tuberculosis (TB) in 2006. The previous eight-month regimen in which rifampicin was administered during the first two months was replaced by a six-month regimen in which rifampicin was given throughout the treatment period. The new regimen has been shown to have greater efficacy, particularly in populations where there is a high prevalence of HIV infection, as it is the case in Tanzania [1], [2]. It is recommended that health care staff directly observe the intake of rifampicin. Daily Directly Observed Treatment (DOT) [3] at health facilities over a six-month period would, however, be unfeasible for both patients and health staff. Patients, most of whom are physically weak, would incur high direct and indirect costs for daily travel, while the workload for health staff would markedly increase and compound the already critical shortage of health care workers. The NTLP therefore introduced an innovative approach which respects the DOT principle, called Patient Centred TB Treatment (PCT). This approach offers new TB patients the choice between home-based treatment (HB-DOT) supervised by a treatment supporter of their own choice, and health facility-based treatment (HF-DOT) observed by a medical professional. The PCT approach was tested for effectiveness in three pilot districts and showed a positive effect on treatment outcome for TB patients [4]. It was adopted as national policy in 2006 and scaled up to the rest of the country. Currently, more than three quarters of all TB patients in Tanzania opt for HB-DOT [5].

However, a potential challenge to the effectiveness of PCT is the difficulty of ensuring adherence to treatment when observation is performed by a non-medical supporter outside a health facility. It is well documented that non-adherence is one of the main contributors to the selection of drug-resistant *Mycobacterium tuberculosis* (*M. tuberculosis*) strains [6]–[8] and subsequent treatment failure [9], [10]. Estimates of the prevalence of drug-resistant strains of *M. tuberculosis* amongst new cases of TB in Tanzania are currently low (1.1%), but data are derived from the conventional eight-month regimen [11].

Adherence to TB treatment in Tanzania has been assessed under HF-DOT or health-worker supervised community-based DOT [12], [13] by indirect measurements including daily clinic attendance, pill counts and sputum-conversion rates. Indirect methods are relatively simple to perform and can be applied to any particular treatment period, but do not provide conclusive measurements of adherence. Pill counts need to be treated with caution due to the recognised practice of pill dumping or pill sharing by patients prior to their scheduled clinic visits [14], [15], while using sputum conversion rates as a proxy for adherence is a somewhat crude approach. Direct assessment of adherence requires the measurement of drug concentrations in the blood or urine and provides proof of intake, but only related to the previous few doses. The necessity of obtaining clinical specimens can preclude its use in an operational setting, but it was undertaken in the pilot study by van den Boogaard *et al* [16] in a region of the highest education levels in the country [17] and confirmed the high level of adherence observed using indirect measurements.

A systematic review of qualitative research has demonstrated that adherence to an extended course of TB treatment under HF-DOT is a complex, dynamic process in which a wide range of factors impact on treatment-taking behaviour [18]. Little is yet known about the factors influencing HB-DOT adherence and an assessment of determinants which foster adherence to TB therapy in this setting would be valuable.

The aim of this study was to assess the extent and determinants of adherence to anti-TB therapy among newly diagnosed patients opting for a home-based regimen under the PCT approach within a large and representative sample of patients in Tanzania.

## Methods

### Study design

This was a cross-sectional study in which information was collected during two stages. In the first stage, patients were visited unannounced at home over a period of six weeks in March and April 2010 to assess their adherence to treatment. In the second stage, during August 2011, information was collected for the same patients with regard to their treatment outcomes, HIV status and use of antiretroviral therapy.

### Ethical statement

The study received ethical approval from the National Research Ethics Committee of the Tanzanian National Institute for Medical Research (NIMR). All participants provided written informed consent to take part in the study.

### Study setting and participants

The study included TB patients from all 93 health facilities providing TB treatment services in four districts of Tanzania. Districts were selected to cover urban and rural locations, former PCT pilot and non-pilot districts, and different areas of the country and comprised Arusha Municipal (urban, pilot), Mufindi (rural, pilot), Mwanza (urban, non-pilot) and Kilosa (rural, non-pilot) districts. In the PCT pilot districts, health workers were extensively trained on how to provide PCT services. Regional and District Tuberculosis and Leprosy Coordinators were trained to perform regular and in-depth supportive supervision. Regular supportive supervision and mentorship was provided by the PCT coordinator and focal person from the central level. In addition, PCT re-training of health workers was undertaken. Finally, communities were sensitised -concerning TB and its care through a social marketing campaign. In the non-PCT pilot districts, no intensive training or sensitisation was conducted and PCT was introduced only as a change to national guidelines to which all health care staff were to comply forthwith, with no regular supportive supervision.

The study population consisted of newly diagnosed TB patients who opted for HB-DOT TB treatment. Patients were eligible to be included in the study if they were aged 18 years or older, currently receiving TB treatment and had provided informed consent to participate.

### Study sample size and sampling procedure

The sample size calculation was based on a single sample estimate of adherence as variable of interest. We compared a postulated estimate for adherence in the intensive phase (85%) with a hypothesized value of adherence in the conventional DOT strategy in Tanzania (75%). The hypothesized population value was based on the conversion rate seen in the control cohort (health facility-based DOT) of the PCT pilot study [4]. The study hypothesis was that the postulated value would not be lower than the hypothesized value, that is, the lower limit of the confidence interval around (CI) the estimate of adherence should be above 75%. The power used was 90%, and the alpha value was set at 0.1 (one-sided testing gives an alpha = 0.05 for falsely rejecting the hypothesis). Based on these parameter values, the number of patients required with a recorded study outcome (adherence to TB therapy) was 137. Allowing for 20% non-consent, the required sample size was 160 patients. This number of patients needed to be included for each of the treatment phases, hence doubling the total sample size to 320. With this sample size, we had 90% power to detect a statistically significant difference between the treatment phases of 15% or more, with alpha = 0.05. To be able to stratify the results with regard to location (urban versus rural) on the one hand, and setting (pilot versus non-pilot) on the other hand, we again doubled the number of patients to be included. This gave us a total sample size of 640 patients.

All public and private health facilities providing TB DOT services in the four districts were visited. In each district we selected 160 new TB patients (80 patients on intensive phase and 80 patients on continuation phase). Simple random sampling was applied, but the number of selected patients varied from facility to facility. This was due to geographical challenges - not all patients could be reached, and hence an unequal number of patients from the individual facilities had to be included to reach the required sample size. When patients could not be reached at home after several attempts, the next patient in the list was selected based on the inclusion criteria.

### Primary outcome and explanatory variables

The primary outcome variable was adherence to tuberculosis therapy as directly measured by a positive IsoScreen assay (GFC Diagnostics Ltd, Bicester, England) [19]. This point-of-care test detects isoniazid and its metabolites in the patient's urine with high sensitivity and specificity [20], confirming whether isoniazid has been taken in the last 24 to 30 hours. It takes only 5 minutes to perform and does not require laboratory facilities, so can be undertaken in the patient's home. The assay was performed according to the manufacturer's instructions. According to Tanzania's TB treatment strategy, patients receive a fixed-dose combination tablet containing both isoniazid and rifampicin. This means that a positive IsoScreen assay result for isoniazid at the same time confirms rifampicin intake.

The following explanatory variables of adherence were investigated: patient gender and age (categorised as 18–24, 25–34, 35–44 and ≥45 years to correspond with national TB recording guidelines), type of TB (sputum smear-positive, sputum smear-negative and extra-pulmonary TB patients), the current treatment phase (intensive or continuation), HIV and antiretroviral therapy status (HIV negative, HIV positive on antiretroviral therapy, or HIV positive not on antiretroviral therapy), whether the patient perceived treatment side effects (yes/no), and the patient's relationship to the treatment supporter (spouse, family member, non-family member). Other explanatory variables of adherence were reported DOT by the treatment supporter on the previous day (yes/no), district location (urban or rural) and setting (pilot or non-pilot district), and the distance between the patient's and the supporter's residence (same house, neighbour, more than 15 minutes walking distance). For logistical reasons, some patients had to be informed of the study team's arrival prior to the home visit, but the purpose of the visit was not communicated. A variable denoting prior contact was therefore included as an additional explanatory variable (yes/no).

### Data collection

In the first stage of data collection, patients were visited at home and interviewed using a structured questionnaire. Patients were asked to provide urine for the IsoScreen assay immediately after the interview while the purpose of the test was explained. Those patients who could not produce urine were asked to drink some water and the interviewer was required to wait for at least one hour to receive a urine sample. Upon receiving the result of the IsoScreen assay, both adherent and non-adherent patients were counselled by the interviewers on the importance of adherence to TB treatment. Furthermore, based on the start date of treatment and the last date of medicine collection, the median week of visiting patients at home was 3rd and 15th week during intensive and continuation phase respectively.

In the second stage of data collection, information on TB treatment outcomes, HIV status and antiretroviral therapy use were obtained from a review of treatment cards and from unit and district TB registers.

Research assistants for data collection were prior trained at the Ifakara Health Institute, Dar es Salaam. Data collection tools (questionnaires and IsoScreen assays) were pre-tested at one of the TB clinics in Dar es Salaam.

Double data entry from collection forms took place at the Ifakara Health Institute. Data were reviewed after entry for out-of-range responses, missing values, or inconsistent skip patterns. The - original data collection sheet was reviewed to resolve any discrepancies or problems. After data entry was completed, the data files were transferred into STATA 12.0 software for analysis (StataCorp LP, College Station, Texas, USA).

### Statistical methods

Patients' characteristics were analysed using descriptive statistics. The question of whether adherence was below 75% was assessed by examining the lower limit of the 90% CI around the point estimate of adherence. A one-sided p-value of 0.05 was deemed statistically significant. We compared adherence in the intensive versus continuation phases, stratified by urban versus rural location, and pilot versus non-pilot setting, using two-sample proportion tests and a two-sided p-value of 0.05 with 95% CI.

The association between adherence and treatment outcome, and between adherence and explanatory variables, was examined using a logistic regression model. Explanatory variables were included in a multivariable model if the association with the primary outcome had a p = value<0.25 on univariable analysis. The baseline multivariable model included at least the variables age, gender and setting (pilot or non-pilot district) based on our theoretical framework. Other variables were fitted to the model using a forward step-wise approach, and assessed by an improvement of the final model based on the pseudo-likelihood ratios and pseudo R^2^ estimates. The collinearity diagnostics were performed among expected interacting variables. All logistic regression analyses contained a probability weighting for each patient to correct for the unequal sampling probability at each facility. This weighting was the inverse of the sampling probability at each facility (1/[number sampled/total TB-patient population at facility]). In addition, the standard errors of the point estimates were corrected for clustering by district.

## Results

### Patient characteristics

**Figure 1:**
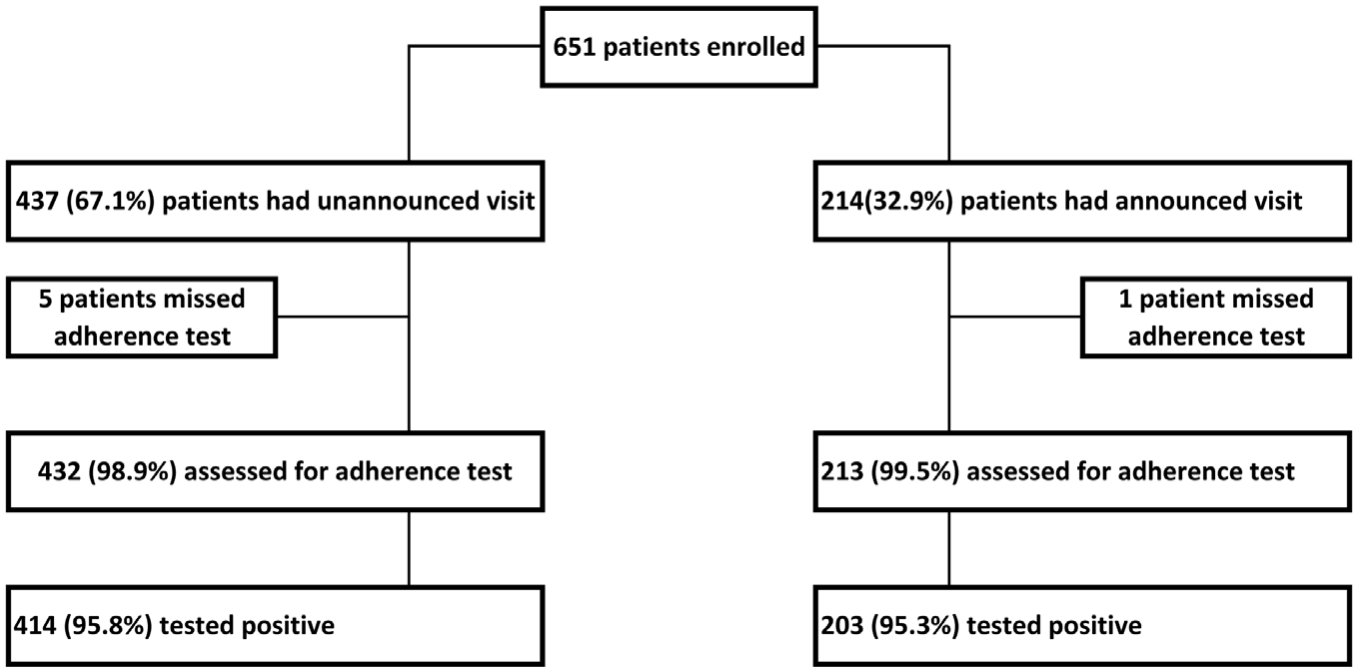
Adherence assessments.

In total, 651 patients were included in the study. Of these, 437 patients (67.1%) received an unannounced visit while 214 patients (32.9%) were contacted in advance by mobile phone. Urine samples were provided by 645 patients (99.1%) (Figure 1). Patient characteristics are summarized in Table 1.

**Table 1:** Characteristics of enrolled patients (N = 651).

Variable	n (%)
**Male**	374 (57.5)
**Age (years)**	
18–24	86 (13.2)
25–34	206 (31.6)
35–44	182 (28.0)
≥45	177 (27.2)
**Type of TB**	
Smear-positive pulmonary TB	294 (45.2)
Smear-negative pulmonary TB	226 (34.7)
Extra-pulmonary TB	131 (20.1)
**HIV infection**	
No	359 (55.1)
Yes	281 (43.2)
Unknown	11 (1.7)
**Setting**	
Non-pilot (Kilosa and Mwanza)	325 (49.9)
Pilot (Mufindi and Arusha)	326 (50.1)
**Location**	
Urban (Arusha and Mwanza)	324 (49.8)
Rural (Kilosa and Mufindi)	327 (50.2)
**Treatment phase**	
Intensive	321 (49.3)
Continuation	330 (50.7)
**Information prior visit**	
Announced	214 (32.9)
Non-announced	437 (67.1)

### Adherence to TB therapy

Of the 645 patients tested, 617 patients (95.7%; 90% CI 94.3%–96.9%) showed a positive assay result, a proportion that was significantly higher than the postulated adherence level of 75% (p<0.001). Adherence to TB therapy was similar for both patients who were and who were not informed of the home visit in advance (95.8% versus 95.3%, respectively; p = 0.757) and for patients in the intensive treatment phase and those in the continuation phase (95.6% versus 95.7%; p = 0.967) regardless of location (urban/rural) or setting (pilot/non-pilot districts). In all subgroups, the lower level of the 90% CI was markedly higher than the postulated value of 75% (Table 2).

**Table 2:** Adherence to anti-TB drugs in intensive and continuation phase (N = 645).

Cohort (N intensive/N continuation)	Intensive			Continuation			
	Adherent n (%)	90% CI	p-value*	Adherentn (%)	90% CI	p-value*	p-value**
Overall (320/325)	306 (95.6)	93.4–97.5	<0.001	311 (95.7)	93.8–97.5	<0.001	0.967
Urban (161/158)	154 (95.7)	93.0–98.3	<0.001	154 (97.5)	95.4–99.5	<0.001	0.374
Rural (159/167)	152 (95.6)	92.9–98.3	<0.001	157 (94.0)	90.9–97.0	<0.001	0.519
Pilot (160/160)	158 (98.8)	97.3–100.2	<0.001	158 (98.8)	97.3–100.2	<0.001	1.000
Non-pilot (160/165)	148 (92.5)	89.1–95.9	<0.001	153 (92.7)	89.4–96.1	<0.001	0.938

* Comparison with hypothesis value of 75% (1-sided 90%CI).

** Comparison between intensive and versus continuation phases (2-sided 95%CI).

Of the 617 adherent patients, 563 (91.2%) successfully completed treatment (i.e. achieved cure or completed the treatment regimen), 19 (3.1%) died, 6 (1.0%) transferred out of the study sites, 2 (0.3%) had treatment failures and 2 (0.3%) defaulted from treatment. Of the 28 non-adherent patients, one patient died while the other 27 patients successfully completed treatment.

### Factors associated with treatment adherence

Patient gender and age were each associated with treatment adherence. In multivariable analysis, female patients were twice as more likely to adhere to treatment compared to male patients (OR: 2.04; 95% CI: 1.24–3.02; p = 0.003). Patients within the 35–44 year age group were less likely to adhere to treatment than those aged 25 years or below (OR: 0.77; 95% CI: 0.59–0.99; p<0.049). Patients with a treatment supporter residing more than 15 minutes walking distance away from their homes were less likely to adhere to treatment than those staying in the same house with the treatment supporter (OR: 0.08; 95% CI: 0.02–0.25; p<0.001). Patients in the pilot districts were almost four times more likely to adhere to treatment than those in non-pilot districts (OR: 3.82; 95%CI: 1.05–13.97; p = 0.043). Treatment adherence was not affected by the type of relationship between the patient and the treatment supporter, the type of TB, HIV status or use of antiretroviral therapy, patient perceptions of treatment-related side effects, location (urban/rural), or whether the treatment supporter had observed the last intake of TB medication (Table 3).

**Table 3:** Crude and adjusted odds ratios for factors fostering treatment adherence among patients on anti-TB drugs (N = 645).

	IsoScreen		Univariate analysis			Multivariate analysis		
	Positive (%)	Negative (%)	OR	95% CI	p-value	OR	95% CI	p-value
**Sex**								
Male	351 (94.9)	19 (5.1)	1	**–**	**–**	1	**–**	**–**
Female	266 (96.7)	9 (3.3)	2.04	0.78–5.30	0.145	1.94	1.24–3.02	0.003
**Age (years)**								
18–24	82 (95.3)	4 (4.7)	1	**–**	**–**	1	**–**	**–**
25–34	196 (96.1)	8 (3.9)	1.69	0.42–6.81	0.457	1.74	0.60–5.01	0.300
35–44	170 (94.4)	10 (5.6)	0.85	0.22–3.28	0.817	0.77	0.59–0.99	0.049
≥45	169 (96.6)	6 (3.4)	1.72	0.41–7.24	0.460	2.15	0.82–5.66	0.120
**Type of TB**								
Smear-negative pulmonary TB	214 (95.1)	11 (4.9)	1	**–**	**–**			
Smear-positive pulmonary TB	279 (95.5)	13 (4.5)	0.95	0.36–2.47	0.919			
Extra pulmonary TB	124 (96.9)	4 (3.1)	2.30	0.65–8.03	0.198			
**Treatment phase**								
Intensive	306 (95.6)	14 (4.4)	1	**–**	**–**			
Continuation	311 (95.7)	14 (4.3)	1.19	0.50–2.83	0.693			
**HIV and antiretroviral therapy status**								
HIV negative	351 (95.9)	15 (4.1)	1	**–**	**–**			
HIV positive on antiretroviral therapy	199 (95.7)	9 (4.3)	0.99	0.37–2.60	0.976			
HIV positive not on antiretroviral therapy	67 (94.4)	4 (5.6)	0.88	0.25–3.18	0.851			
**Perceived side effect**								
No	434 (95.8)	19 (4.2)	1	**–**	**–**			
Yes	182 (95.3)	9 (4.7)	0.98	0.89–1.01	0.785			
**Relationship of supporter**								
Non-family member	187 (96.4)	7 (3.6)	1	**–**	**–**			
Family member	203 (94.4)	12 (5.6)	0.70	0.24–2.13	0.538			
Spouse	202 (96.7)	7 (3.3)	1.62	0.48–5.50	0.437			
Missing	25 (92.6)	2 (7.4)	0.75	0.14–4.18	0.747			
**Residence of supporter**								
Same house	471 (95.9)	20 (4.1)	1	**–**	**–**	1	**–**	**–**
Neighbour	131 (97.0)	4 (3.0)	1.65	0.49–5.57	0.420	1.38	0.77–2.47	0.284
>15 minutes walk	15 (78.9)	4 (21.1)	0.08	0.022–0.29	<0.001	0.08	0.02–0.25	<0.001
**Information prior to the visit**								
Announced	414 (95.8)	18 (4.2)	1	**–**				
Non-announced	203 (95.3)	10 (4.7)	0.86	0.36–2.09	0.744			
**Last dose observed by supporter**								
No	138 (91.4)	13 (8.6)	1	**–**	**–**			
Yes	479 (97.0)	15 (3.0)	1.09	0.99–1.19	0.053			
**Setting**								
Non-pilot	301 (91.6)	24 (7.4)	1	**–**	**–**	1	**–**	**–**
Pilot	316 (98.8)	4 (1.2)	3.82	1.05–13.97	0.043	3.65	0.65–20.39	0.141
**Location**								
Urban	308 (96.6)	11 (3.4)	1	---	**–**			
Rural	309 (94.8)	17 (5.2)	0.95	0.86–1.05	0.302			

## Discussion

These results indicate that 95% of TB patients who opted for home-based treatment under the PCT approach adhered to the treatment regimen. This was markedly higher than the levels of adherence reported from other studies conducted in Uganda (75%) [21], South Africa (85%) [22] and Ethiopia (79.2%) [23]. Home-based treatment under a PCT approach was not associated with increased non-adherence during the continuation phase, in contrast to conventional daily HF-DOT in which adherence tends to decline after the intensive treatment phase when symptoms have resolved [22], [24].

Overall, adherence to HB-DOT with PCT was higher in pilot districts than in non-pilot districts. In some of the health facilities of the Kilosa non-pilot district there were stock outs of anti-TB drugs during the data collection period, which would be expected to have contributed to the lower rate of adherence compared to the other non-pilot district (Mwanza). However, the effect of the district setting (pilot/non-pilot) persisted when analyses were repeated omitting Kilosa district. Improved adherence in pilot districts may be attributed to more intense training of health care workers as well as the regular supportive supervision and mentoring provided throughout the pilot phase. In the non-pilot districts, training was given only once, with no such regular supervision or mentoring. In addition, TB social marketing campaigns were conducted only in the pilot districts and may have created greater community awareness, potentially contributing to higher utilisation of TB services and adherence rates [25]. These types of interventions have been shown to improve the quality of TB services and control in other African settings [26]–[28].

Adherence in patients whose last dose intake had been observed by the treatment supporter was similar to that in patients whose last drug intake had not been observed. It has been reported that patients who are directly observed during drug intake adhere to TB treatment [29], [30], but our results suggest that observation of drug intake does not need to be on a daily basis. It could be argued that regular motivation and support is sufficiently adequate, and that a daily formal observation does not contribute to increased levels of adherence. However, this does not suggest that support and observation should be abolished all together. Of interest, patients with a treatment supporter living in the same home or a neighbouring house showed greater adherence than those with a supporter who lived further away. This contradicts earlier findings from the PCT pilot study, which found that no supporter-related factors affected the treatment outcome of the patient [4] although the effect of the supporter's location could not be examined due to the strong collinearity between the type of supporter (family member versus non-family member)and location of the supporter. It appears that close community support, for example from family members, is very important during TB treatment. It has previously been documented that DOT supervised by a family member is an effective and low-cost technique [4], [31], [32].

Our data show that non-adherence can be corrected and does not necessarily lead to poor treatment outcome. The few patients who were non-adherent on the day of the IsoScreen assay nonetheless successfully completed treatment, possibly as a consequence of home counselling by the study team immediately after the IsoScreen testing. This confirms the conclusion of other studies that counselling of TB patients about treatment adherence can promote treatment completion [33]–[36].

It was noteworthy that female TB patients showed higher adherence to the treatment regimen than males, consistent with the results of a study from Thailand in which males had a lower success rate than females [37]. This suggests that targeted efforts - through education campaigns and counselling by healthcare providers – are required to improve awareness among men of the importance of TB treatment adherence.

The current study has several limitations. Data on adherence were collected in the frame of a cross-sectional survey which did not permit an analysis of adherence over time in the same patients, as can be undertaken using a cohort design. This meant that adherence in the intensive and continuation treatment phases had to be assessed in different patients. However, the high rates of treatment success confirmed the study participants' adherence to TB therapy. The study faced potential ascertainment bias by the necessity of announcing the home visit to a third of study participants in advance either because address details were missing on the treatment card or because the interviewers could not locate the patients. This might have prompted drug intake among these participants before the visit However, since the patients were not informed of the purpose of the visit a pronounced effect seems unlikely, as indicated by the similar of adherence rates among patients who were and those who were not notified in advance.

Strengths of the study were that it included a large and varied group of patients from different settings and locations, and that it was performed under programmatic conditions. The study sample was representative of Tanzania as a whole and may be relatively generalisable to other countries with similar characteristics although stepwise implementation of the PCT approach with careful evaluation is recommended in other settings. It may be useful to tailor this patient-centred HB-DOT approach to control programmes for other chronic diseases where long-term adherence to home-administered drugs is paramount, be it prophylactic drugs (e.g. cotrimoxazole prophylaxis in HIV patients and nevirapine prophylaxis in breastfed infants of HIV-positive mothers) or therapeutic drugs (e.g. antiretroviral therapy in HIV patients, anti-diabetic medication and antihypertensive drugs).

In conclusion, evidence from this study suggests that HB-DOT using a PCT approach can achieve high rates of adherence and treatment success among TB patients. However, a reliable supply of medication and the careful selection of treatment supporters, who preferably live close to the patient, are crucial success factors. Finally, we recommend a cohort study to assess the rate of adherence throughout the full course of TB-treatment.
